# A complex system health state assessment method with reference value optimization for interpretable BRB

**DOI:** 10.1038/s41598-024-52829-3

**Published:** 2024-01-28

**Authors:** Qingxi Zhang, Kangle Li, Guangling Zhang, Hailong Zhu, Wei He

**Affiliations:** 1 School of Computer Science and Information Engineering, Harbin Normal University, Harbin, 150025 China; 2 Harbin Finance University, Harbin, 150030 China

**Keywords:** Computer science, Engineering

## Abstract

Health condition assessment is the basis for formulating and optimizing maintenance strategies of complex systems, which is crucial for ensuring the safe and stable operation of these systems. In complex system health condition assessment, it is not only necessary for the model to handle various uncertainties to ensure the accuracy of assessment results, but also to have a transparent and reasonable assessment process and interpretable, traceable assessment results. belief rule base (BRB) has been widely used as an interpretable modeling method in health condition assessment. However, BRB-based models currently face two issues: (1) inaccuracies in expert-provided parameters that can affect the model's accuracy, and (2) after model optimization, interpretability may be reduced. Therefore, this paper proposes a new method for complex system health condition assessment called interpretable BRB with reference value optimization (I-BRB). Firstly, to address the issue of inaccurate reference values, a reference value optimization algorithm with interpretability constraints is designed, which optimizes the reference values without compromising expert knowledge. Secondly, the remaining parameters are optimized using the projection covariance matrix adaptation evolution strategy (P-CMA-ES) with interpretability constraints to improve the model's accuracy. Finally, a case study evaluating the bearing components of a flywheel system is conducted to validate the proposed method. Experimental results demonstrate that I-BRB achieves higher accuracy in health condition assessment.

## Introduction

For critical complex systems such as aerospace and nuclear power plants, safety and reliability are of paramount importance^1^. Health condition assessment plays a crucial role in identifying potential safety hazards and risks, allowing for timely intervention and repair to ensure the safe operation of complex systems and protect the well-being of personnel and the environment^2^. Researchers have conducted extensive studies in this field, achieving fruitful outcomes. For example, in the health assessment of complex and large-scale civil structures, Daneshvar et al. proposed a novel machine learning approach for unsupervised information-driven structural health monitoring anomaly detection in both long-term and short-term monitoring applications in civil engineering^3^. Entezami et al. proposed a novel method for early damage detection in large-scale bridge structures under long-term monitoring^4^. Alarcón et al. proposed a low-cost seismic instrumentation system (LCSIS) for monitoring the structural health of South America's first experimental 6-story light-frame timber building^5^. Chen et al. addressed the issue of continuous missing data in the health diagnosis of concrete dams by proposing and validating a health diagnosis model based on domain learning^6^. In the assessment of the health status of complex systems, reliable and interpretable evaluation results enhance the persuasiveness of the assessment^7^. Based on reliable and interpretable assessment results, decision-makers can better understand the health status of the system and the existing risks, enabling them to take timely measures for intervention and repair^8^. This ensures the safe operation of the system and safeguards the well-being of personnel and the environment^9^. Moreover, the assessment results provide essential information for decision-makers regarding maintenance and update plans, further enhancing the safety and reliability of the system^10^.

Interpretability refers to the model's ability to express the behavior of a system in an understandable manner^11^. It is a subjective and open concept that requires further discussion. Many scholars have conducted in-depth research on the interpretability of models, but due to the subjective nature of understanding interpretability, a unified definition has not yet been established^12^. Different researchers approach the issue from various perspectives, assigning different meanings to interpretability, and consequently, proposed interpretability methods may also have different emphases^7^. With the increasing demand for reliability in practice, establishing models that are both reliable and interpretable has become a crucial objective in enhancing human understanding of real-world systems^11^. In current research on health condition assessment for complex systems, the constructed evaluation models can be broadly categorized into three types: (1) Black-box models: These models are data-driven and their internal workings and decision-making processes are opaque to users or observers^13^. Users can only observe the inputs and outputs of the model without knowing the specific details and reasoning process^14^. Therefore, the evaluation results obtained from this approach are challenging to be acknowledged by decision-makers^15^. (2) White-box models: These models are typically built based on system mechanics and fully simulate the system's operation process^1^. White-box models are typically used to describe systems with explicit rules, parameters, and logic. The transparency of such models allows users to delve into every component of the model, understanding how each part processes inputs, makes decisions, and generates outputs. The modeling process and inference results of white-box models are interpretable^16^. However, accurately analyzing the interactions among various components of complex systems poses significant challenges^17^. Therefore, constructing a reasonable and effective white-box model is highly difficult^18^. (3) Gray-box models: These models combine the advantages of black-box and white-box models by integrating model inference and data sample construction, maintaining a certain level of accuracy and interpretability^11^. Based on these characteristics, gray-box models have been widely applied in health condition assessment research^12^.

In complex systems, the use of data-driven models requires a large number of data samples to build accurate evaluation models. However, due to the characteristics of complex systems such as high value and short lifecycle, acquiring sufficient data samples can be challenging^2^. This limitation restricts the application of traditional data-driven models. It is worth noting that in the field of health condition assessment for complex systems, expert knowledge becomes particularly important due to the limited amount of data^19^. Furthermore, issues arising in these high-risk complex systems can potentially lead to severe economic or even strategic costs, often demanding a high level of credibility for the assessment models^11^. Therefore, the modeling process of complex systems should be reliable and transparent, allowing decision-makers to comprehend it for formulating trustworthy decisions^20^. The comprehensive utilization of quantitative data and qualitative knowledge can effectively address the challenges of health status assessment in complex systems, particularly in scenarios involving limited sample sizes^21^. Experts have accumulated rich experience and knowledge through long-term practice and can provide valuable insights into system behavior, performance, and health condition^22^. BRB is a gray-box model that effectively utilizes small-scale data from engineering practice and combines expert knowledge, demonstrating strong modeling capabilities^22^. BRB is a non-linear modeling method that can express various forms of uncertain information, including randomness and ignorance. Moreover, BRB is a modeling approach based on IF–THEN rules with strong causal reasoning capabilities. The interpretability modeling of BRB can be mainly divided into three parts: pre-modeling, in-modeling, and post-modeling interpretability^11^. (1) Pre-modeling interpretability refers to the interpretability obtained by experts through the analysis of the actual system mechanisms or long-term work practices. Rule-based modeling methods, such as BRB, can extract rules from expert knowledge, making the model easy to understand. (2) In-modeling interpretability refers to the transparency of the inference process. Rule-based modeling methods, including BRB, primarily use techniques like fuzzy reasoning and approximate reasoning for computation. Rules in BRB are a series of explicit logical statements, typically in the form of if–then^11^. This clear and intuitive structure allows people to understand the meaning of the rules and what happens under specific conditions. Rules are often expressed in natural language or other easily understandable forms^20^. (3) Post-modeling interpretability refers to attempting to interpret the workings of the model after the training process is completed. Due to its strong causal reasoning ability, rule-based modeling methods like BRB enable traceability of the model's output results^12^. Therefore, BRB is highly suitable for health condition assessment in complex systems. This approach, which integrates the advantages of data-driven models and white-box models, can provide reliable and interpretable evaluation results^20^.

In current research on constructing complex system health condition assessment models based on BRB, the parameters of the model are predefined by experts^23^. However, due to the subjectivity and limitations of expert knowledge, the initial model built is not precise, which can affect the assessment effectiveness^24^. To enhance its modeling capabilities, researchers have conducted extensive studies. For example, Feng et al. proposed a safety assessment model based on BRB-r, which considers the reliability of belief rules to balance the complexity and accuracy of the model^8^. Sun et al. introduced a new type of BRB called BRB-IR, which incorporates qualitative knowledge and quantitative data with interval-valued references to construct the model^16^. These studies have expanded the modeling approaches of BRB and improved its modeling capabilities to some extent.

However, there are still two issues with the current complex system health condition assessment models based on BRB^25^. Firstly, due to the subjectivity and limitations of expert knowledge, the parameters provided by experts for constructing BRB-based health condition assessment models may not be accurate enough, which can affect the accuracy of the assessment results^26^. The existing BRB-based health condition assessment methods mainly focus on optimizing the belief degrees, attribute weights, and rule weights of BRB, but the optimization of reference values is rarely considered, limiting the accuracy of the models^27^. Secondly, during the optimization of BRB-based health condition assessment models, the interpretability of the models may be compromised. Therefore, to address these issues, this paper proposes a new method for complex system health condition assessment, which incorporates reference value optimization into an interpretable BRB framework.

The contributions of this paper are as follows: (1) The introduction of the I-BRB method. This method enables the evaluation of complex system health conditions in an interpretable manner. By incorporating reference value optimization, it enhances the accuracy of the assessment results. (2) A novel reference value optimization method with interpretability constraints. To further improve the accuracy of I-BRB, a new approach is proposed to optimize the reference values while maintaining interpretability. This method addresses the issue of inaccurate parameters provided by experts and ensures the reliability of the assessment process. (3) The design of interpretability constraints for complex system health condition assessment. In the context of assessing complex system health conditions, interpretability constraints are introduced to preserve the interpretability of the models during the optimization process. This constraint ensures that the models remain transparent and explainable, facilitating the understanding and acceptance of the assessment results.

The remaining structure of the paper is organized as follows: In Section "Problem description", attention is directed towards three critical issues that need consideration when constructing models for the assessment of health conditions in complex systems. Emphasis is placed on outlining the challenges and prerequisites associated with accuracy, interpretability, and reference value optimization. In Section "Basic BRB and interpretability definitions", the basic BRB model is introduced, accompanied by a definition of interpretability. Fundamental concepts of BRB are explained, setting the foundation for the subsequent development of the I-BRB model. In Section "Inference and optimization", a reference value optimization algorithm is proposed. Detailed descriptions of the inference and optimization processes within the I-BRB model for assessing the health condition of complex systems are provided. The algorithm incorporates interpretability constraints to ensure the accuracy and interpretability of the evaluation results. In Section "Case study", a case study is presented, focusing on the health condition assessment of an aerospace engine flywheel system. This case study serves as a validation of the effectiveness and performance of the proposed I-BRB method in a practical application scenario. In Section "Conclusion", the paper concludes with a summary of the key findings and contributions of the research. Furthermore, potential directions for future work are discussed, and the significance of the proposed I-BRB method in the context of complex system health condition assessment is considered.

## Problem description

To construct an interpretable I-BRB model for complex system health assessment, three key issues need to be addressed:

### Problem 1:

How to guarantee interpretability in complex system health state assessment models? Considering the characteristics of complex systems and the requirements of health state assessment, there is a need to design reasonable interpretability constraints to maintain the interpretability of the whole modelling, inference, and optimisation process^23^. This process could be described as follows:

1 $$ Interpretability:\{ C\left| {C_{1} } \right.,C_{2} , \ldots ,C_{z} \} $$

where $C$ is the set of interpretable constraints, $z$ represents the number of interpretability constraints.

### Problem 2:

How to construct a transparent reasoning process that meets the interpretability requirements of complex system health state assessment? In building the initial BRB model for complex system health state assessment, it is important to consider parameter settings and the rationality of the reasoning process in order to maintain the interpretability of the inference results. This process can be described as follows:

2 $$ s = f(data,t,C,ek) $$

where $s$ denotes the final belief distribution, $data$ denotes the set of evaluation indicators for health state assessment, $t$ denotes the initial parameters given by the experts, and $ek$ denotes the expert knowledge, $f( \cdot )$ denotes the inference function.

### Problem 3:

How to improve the accuracy of the model without compromising its interpretability? Optimizing the parameters of the complex system health state assessment model can further enhance its accuracy^11^. It is therefore important to design a rational optimisation process that takes into account the interpretability constraints of the model. The interaction between the interpretability constraint and the optimisation process can be described as follows:

3 $$ t_{best} = optimize(data,\Omega ,s,C,) $$

where $\Omega$ denotes the set of parameters in the optimization process.

## Basic BRB and interpretability definitions

### Basic BRB

The BRB model is based on the IF–THEN modeling approach and consists of multiple rule^28^. The $k_{th}$ rule in the model can be expressed as follows:

4 $$ \begin{gathered} R_{k} : \hfill \\ if \, x_{1} \, is \, RA_{1}^{k} \wedge \, x_{2} \, is \, RA_{2}^{k} \wedge \cdots \wedge \, x_{T} \, is \, RA_{T}^{k} \hfill \\ then\{ (D_{1} ,\beta_{1,k} ),(D_{2} ,\beta_{2,k} ), \cdots ,(D_{N} ,\beta_{N,k} )\} ,(\sum\limits_{n = 1}^{N} {\beta_{n,k} \le 1} ), \hfill \\ with \, rule \, weight \, \theta_{k} ,k\{ 1,2, \cdots ,L\} . \hfill \\ and \, attribute \, weight \, \delta_{1} ,\delta_{2} , \cdots \delta_{i} ,i \in \{ 1,2, \cdots ,W\} \hfill \\ \end{gathered} $$

where $x_{i} (i = 1,2, \cdots ,T)$ represents the $i_{th}$ indicator of the complex system health assessment, $RA_{i}^{k}$ is the reference value provided by experts for the $i_{th}$ evaluation indicator, $D_{i} (i = 1,2, \cdots ,N)$ represents the $i_{th}$ evaluation result, $\beta_{1,k} ,\beta_{2,k} , \cdots ,\beta_{N,k}$ represents the belief level of each evaluation result under the $k_{th}$ rule, $\theta_{k}$ represents the weight of the $k_{th}$ rule, and $\delta_{i}$ represents the attribute weight of the $i_{th}$ attribute.

### Interpretability definitions

**Figure 1:**
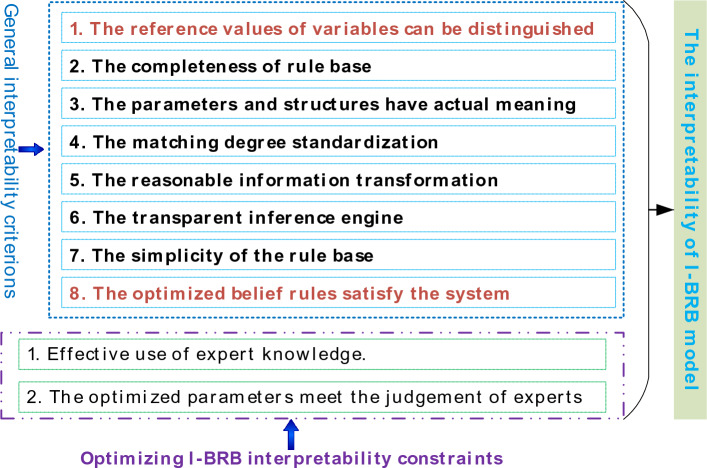
Interpretability criterions of I-BRB.

The importance of understanding and interpreting assessment results in complex system health assessment cannot be ignored. Decision-makers need to understand the basis and reasoning process of assessment results in order to make informed decisions and take appropriate actions. Therefore, to maintain the interpretability of the I-BRB model, it is necessary to establish a reasonable and effective definition of interpretability. In reference^11^, a set of general interpretability criterion for BRB was designed and defined, and I-BRB conforms to these general interpretability criterions. Additionally, addressing the existing issues in current BRB-based complex system health assessment models, this paper specifically emphasizes criterions 1 and 8. The I-BRB interpretability criterions is illustrated in Fig. 1.

*Criterion 1*: The reference values of variables can be distinguished.

In BRB, the reference values represent the positions on the evaluation scale where an attribute has typical meanings^19^. They should be able to differentiate different ranges of the variable space and are typically set by experts based on domain knowledge and experience. The setting of reference values should match the specific implementation objectives and application scenarios, as different domains may require different approaches for setting reference values. Therefore, it is important to reasonably divide the reference value intervals for the evaluation indicators of complex system health status and assign them to different ranges of evaluation levels. These ranges should not overlap, and the reference value ranges should encompass the meanings associated with the evaluation indicators, ensuring a clear distinction between different divisions to meet the requirements of real complex systems.

Due to the significant uncertainty in complex systems, the reference values provided by experts may not be precise enough. This could impact the accurate differentiation of system states and, consequently, hinder the understanding of the system^12^. Additionally, it may limit the accuracy of the complex system health condition assessment model. Typically, reference values for technical indicators in a system can exist within a certain range. When constructing a BRB, the reference values provided by experts are often empirical values within a feasible range, rather than exact values. Therefore, to enhance the accuracy of the I-BRB model without sacrificing interpretability, it is necessary to optimize the reference values within a reasonable range. The optimal reference values should be determined within the feasible interval provided by experts, and this can be described as:

5 $$ \begin{gathered} A_{i}^{k} \sim Q_{i}^{k} \left( {k = 1,2, \cdots ,L} \right) \hfill \\ s.t. \hfill \\ h \in t \hfill \\ Q_{k} \in \left\{ {\left\{ {RA_{i}^{k} \_Min \le A_{i}^{k} \le RA_{i}^{k} \_Max} \right\}} \right\} \hfill \\ h \in \{ \left\{ {A_{1}^{k} < A_{2}^{k} < \cdots < A_{i}^{k} } \right\} \hfill \\ or\left\{ {A_{1}^{k} > A_{2}^{k} > \cdots > A_{i}^{k} } \right\}\} \hfill \\ \end{gathered} $$

where $Q_{i}^{k}$ represents the interpretability constraint for the $i_{th}$ reference value in the $k_{th}$ rule, $RA_{i}^{k} \_Min$ and $RA_{i}^{k} \_Max$ denote the maximum and minimum acceptable values for the reference value as determined by the experts, $h$ represents the set of reference values. This constraint ensures that the optimized reference values remain within the acceptable physical range during the reference value optimization process. By doing so, it prevents the parameters from deviating too far from the initial values provided by the experts, thus preserving the influence of expert knowledge.

*Criterion 8*: The optimized rules satisfy the requirements of complex system health state assessment.

In complex system health state assessment using I-BRB, it is essential that each step can be clearly described, and there should be a reasonable cause-and-effect relationship between the inputs and outputs. This is a prerequisite to ensure that the results of the assessment are understood and accepted for decision makers^29^. In the construction of an I-BRB-based model for assessing the health status of complex systems, the expert knowledge is translated as parameters as well as applied to the construction of rules. Therefore, the model's inference results possess interpretability. However, in practical engineering problems, optimisation algorithms are often used to enhance model assessment accuracy. The use of optimisation algorithms to optimise model parameters is stochastic, which can undermine expert knowledge and lead to unconvincing evaluation results.

For example, in the assessment of the health state of an aircraft engine, the belief distribution of the output results is given as {(excellent: 0.35) (good: 0.1) (fair: 0.1) (poor: 0.45)}. This implies that the probability of the aircraft engine being in an excellent health state is 0.35, and the probability of it being in a poor health state is 0.45. Clearly, such an assessment result is unreasonable. The correct assessment result should be able to reasonably differentiate between two conflicting health states^30^.

**Figure 2:**
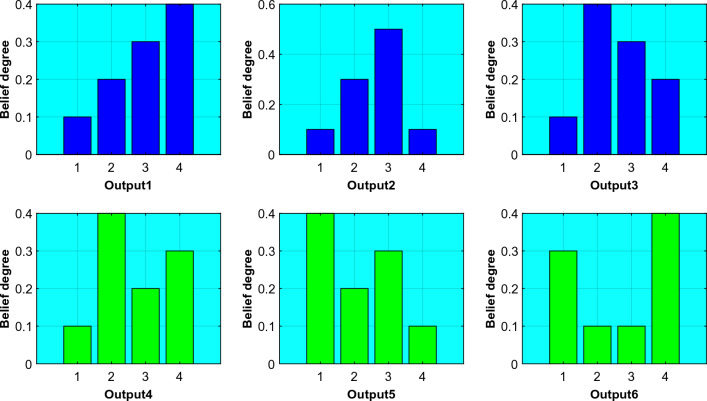
Reasonable belief distribution vs. Unreasonable belief distribution.

Therefore, in order to ensure that the initial expert knowledge is not disrupted during the optimization process of the model, the following interpretability constraints are proposed:

6 $$ \begin{gathered} \beta_{k} \sim Z_{k} \left( {k = 1,2, \ldots ,L} \right) \hfill \\ Z_{k} \in \{ \left\{ {\beta_{1} \ge \beta_{2} \ge \cdots \ge \beta_{n} } \right\} \hfill \\ \vee \left\{ {\beta_{1} \le \beta_{2} \le \cdots \le \beta_{n} } \right\} \hfill \\ \vee \left\{ {\beta_{1} \le \beta_{2} \le \cdots \le \max \left( {\beta_{1} ,\beta_{2} , \ldots ,\beta_{n} } \right) \ge \ldots \ge \beta_{n} } \right\}\} \hfill \\ \end{gathered} $$

where $Z_{k}$ represents the interpretability constraint in the $k_{th}$ rule, which may vary depending on different system characteristics. However, they should all satisfy the actual belief distribution. A reasonable belief distribution shape should be monotonic or convex. As shown in Fig. 2, the belief distributions of Output1, Output2, and Output3 are reasonable. On the other hand, the belief distributions of Output4, Output5, and Output6 are concave or non-monotonic, which clearly indicates conflicting belief distributions^11^.

Complex system health assessment models constructed on the basis of BRB have traceable relationships between inputs and outputs, which makes the interpretability of the model an inherent feature. However, due to limited expert knowledge, experts build initial models that may not meet the requirements of the actual system and require optimisation using observed data^28^. Nevertheless, algorithms for optimisation introduce stochasticity, and this can compromise the interpretability for health assessment models. Given the stringent reliability requirements for health assessment results of complex systems, in order to maintain the interpretability of the BRB model, the following constraints were designed.

*Constraint 1*: Effective use of expert knowledge.

Domain experts typically possess rich knowledge and experience, providing them with a deeper understanding of the problem domain^11^. The complex system health assessment model based on BRB effectively incorporates this valuable expertise and insights into the model, thereby enhancing its accuracy and predictive capabilities. This becomes an important source of interpretability for the BRB-based model. The process of optimisation in the interpretable BRB model is based as a local search guided by initial expert judgement^17^. Thus, of expert knowledge is translated and incorporated in the initial population for the optimisation algorithm, providing instructions for the optimisation process and efficiently extracting useful pieces of information out of the search space.

7 $$ w^{g} = \left\{ {\begin{array}{*{20}c} {ek, \, if \, g = 1} \\ {w^{g} , \, if \, g \ne 1} \\ \end{array} } \right. $$

where $w^{g}$ represents the parameters of the $g_{th}$ generation.

*Constraint 2*: The optimized parameters meet the judgement of experts.

In complex system health assessment, the interpretability of the evaluation results is of paramount importance. When constructing a health assessment model using BRB, the parameters are derived from expert knowledge^11^. Compared to black-box models, the evaluation results of BRB have interpretability and can be convincing to decision-makers. However, when optimizing the BRB model using optimization algorithms, it is possible for the parameters to lose their original meanings and deviate significantly from the initial expert knowledge. This can make the evaluation results difficult to trust. To address this issue, it is possible to set reasonable range constraints to ensure that the parameters vary within an acceptable physical range. This can prevent the parameters from deviating too far from the initial values provided by the experts and preserve the influence of expert knowledge. Therefore, the proposed interpretability constraints are as follows:

8 $$ \begin{gathered} H_{lp} \le H \le H_{up} :\{ \theta_{{lp_{k} }} \le \theta_{k} \le \theta_{{up_{k} }} \, k \in \{ 1,2, \ldots ,L\} . \hfill \\ \delta_{{lp_{i} }} \le \delta_{i} \le \delta_{{up_{i} }} \, n \in \{ 1, \ldots ,N\} . \hfill \\ \beta_{{lp_{k,n} }} \le \beta_{k,n} \le \beta_{{up_{k,n} }} \, i,n \in \{ 1,2, \ldots ,T\} \} . \hfill \\ \end{gathered} $$

where $H_{lp}$ and $H_{up}$ denote the lower and upper bounds of the parameters, respectively. The parameters referred to here include rule weights, attribute weights, and belief degrees.

In the context of complex system health assessment based on BRB, the model's rules are constructed based on the knowledge and expertise of domain experts. Each rule describes a specific decision or reasoning process under certain conditions^30^. These rules can be obtained through interactions with domain experts, knowledge extraction, or rule learning techniques. The parameters in the BRB model have practical meanings and can be interpreted as weights and belief degrees assigned to rules and conditions. Furthermore, the inference process of the BRB model is interpretable, as the model can demonstrate how it performs reasoning and decision-making based on input conditions and rules^9^. By tracing the inference process, users can understand the logical reasoning and basis behind the model's decisions. Such interpretability allows users to comprehend the decision-making logic and rationale of the model. These characteristics make the BRB model widely applicable in complex system health assessment, particularly in application scenarios where model interpretation and understanding are essential. To optimize the model without compromising its interpretability, it is necessary to introduce the aforementioned interpretability constraints.

## Inference and optimization

### Reference value optimization

Complex systems often have numerous variables and interconnected parameters, and their operating mechanisms can be complex and partially unknown. Due to the system's uncertainty, experts may have limitations in understanding the system, resulting in less accurate reference values. Furthermore, the provision of expert knowledge is often influenced by individual subjectivity and experience. Different experts may have varying viewpoints and preferences, leading to differences in the reference values they provide. In some cases, experts may also face the challenge of insufficient data. Particularly in emerging fields or complex system assessments, the available data may be limited, affecting the experts' ability to provide accurate reference values.

The accuracy of the complex system health assessment model based on BRB is influenced by the reference values, as even slight differences in reference values can impact the assessment results. Setting reference values should be meaningful and aim to activate as many rules as possible. Due to the uncertainty of complex systems, the reference values provided by experts may not be precise^22^. This can impact the differentiation of system states and further affect the understanding of the system. Typically, reference values for technical indicators of a system can be a range of values. In the BRB, reference values represent the range of values for rule antecedent attributes, used to transform input data into belief distributions and support the calculation of rule activation weights^7^. The selection of reference values is crucial as it significantly influences the performance of the model. Firstly, reference values should cover all possible ranges of rule attributes. This ensures that input data falls within the range of some reference value, enabling reasonable membership degree calculations. This is critical because if reference values cannot cover the entire range of possible values, it will lead to inadequate reasoning for all input data^23^. Additionally, the design of reference values should minimize overlapping regions as much as possible^20^. This means that the intersection between different reference values should be kept minimal to avoid situations where input data has high membership degrees in multiple reference values, causing uncertainty in rule activation weights. Reducing intersections helps improve the stability of system decision-making. Therefore, it is necessary to optimize the reference values without compromising the model's interpretability.

**Figure 3:**
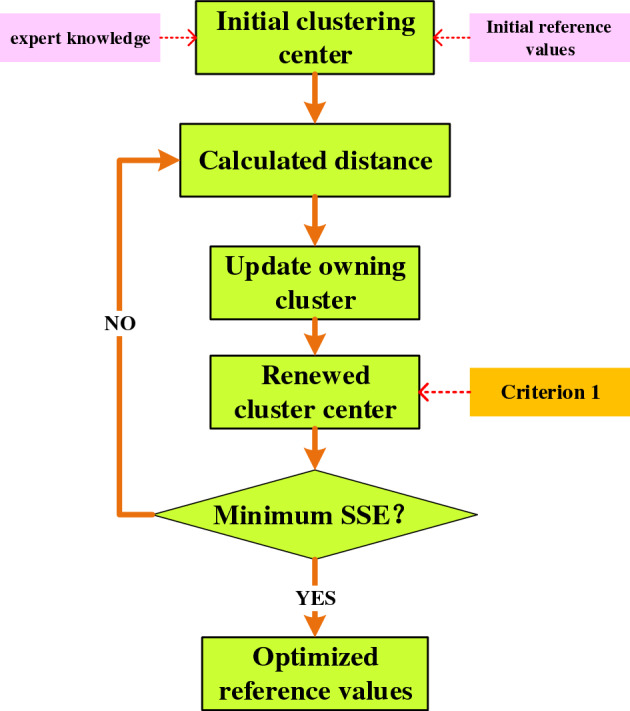
Reference value optimization algorithm.

Based on the above analysis, this paper proposes a K-means algorithm with interpretability constraints (KA-WIC), as shown in Fig. 3. To preserve the model's interpretability, this paper introduces certain constraint conditions in KA-WIC to guide the optimization process of the reference values. Firstly, to effectively utilize expert knowledge, the reference values provided by experts are used as the initial cluster centers. This ensures that the optimization process starts from a meaningful and expert-guided initialization point. Secondly, the optimization process incorporates the experts' prior knowledge or experience as additional constraint conditions. This helps to enforce the rationality and accuracy of the reference values under the guidance of expert knowledge. By integrating these interpretability constraints into the optimization process, the proposed approach ensures that the reference values are optimized while maintaining the interpretability of the model. This allows for a more accurate and reliable assessment of the complex system's health status, leveraging both expert knowledge and data-driven optimization techniques.

By incorporating these interpretability constraints into the K-means algorithm, it is possible to consider both the data characteristics and expert knowledge during the optimization process of the reference values, without compromising the model's interpretability. This ensures that the optimized reference values are more aligned with the actual requirements and are easier to interpret and understand. It is important to note that when introducing constraint conditions, a balance between interpretability and clustering performance needs to be struck to ensure the effectiveness and accuracy of the algorithm.

The KA-WIC algorithm clusters data points by minimizing the distance between data points and cluster centers. Therefore, each cluster's center represents the data points within that cluster. The cluster center can be seen as the average or centroid of the data points within the cluster, as they are close in proximity to other data points and exhibit higher similarity. Thus, using the cluster center as a reference value provides a holistic description of the overall characteristics of the data within that cluster.

Moreover, cluster centers can also be seen as a summary of the data distribution. By calculating the coordinates or feature values of the cluster centers, we can obtain the average or central tendencies of the data in each dimension. These tendencies can reveal the concentration, bias, or focus of the data in different dimensions. Therefore, using cluster centers as reference values provides an understanding of the overall data distribution, aiding in the comprehension of data concentration and distribution patterns.

In order to optimize the reference values of the model, the objective function is formulated as follows:

9 $$ h = (A_{1}^{1} ,A_{1}^{2} , \cdots ,A_{i}^{k} ) = oa(RA_{1}^{1} ,RA_{1}^{2} , \cdots ,RA_{i}^{k} ,C),h \in t $$

where $RA_{i}^{k}$ represents the $k_{th}$ reference value for the $i_{th}$ attribute given by the expert, $A_{i}^{k}$ represents the $k_{th}$ optimized reference value for the $i_{th}$ attribute, and $oa( \cdot )$ denotes the interpretability-optimized algorithm for reference value mining. The detailed steps of the KA-WIC algorithm for mining the reference value set are as follows:

*Step 1*: Initialize the reference value set A by using the expert-provided reference values as the initial cluster centers.

10 $$ \begin{gathered} \mu_{1} = RA_{1}^{1} ,\mu_{2} = RA_{2}^{1} , \cdots ,\mu_{i} = RA_{i}^{k} ,i \in [1,T] \hfill \\ c_{1} ,c_{2} , \cdots ,c_{i} \leftarrow \mu_{1} ,\mu_{2} , \cdots ,\mu_{i} \hfill \\ \end{gathered} $$

where, $c_{i}$ represents the $i_{th}$ cluster, $\mu_{i}$ represents the $i_{th}$ cluster center, and $T$ represents the number of cluster centers.

*Step 2*: Calculate the Euclidean distance between two points. For each data point and each cluster center, calculate the distance between them as follows:

11 $$ dist(x_{j} ,u_{i} ) = \left\| {x_{j} - u_{i} } \right\|^{2} $$

12 $$ x_{j} = d_{j} \in data $$

where $d_{j}$ and $x_{j}$ represent the $j_{th}$ data point of the health assessment indicator $data$, $M$ represents the total number of data points, and $dist(x_{j} ,u_{i} )$ is used to denote the distance from data point $x_{j}$ to cluster center $u_{i}$.

*Step 3*: Update the assigned cluster for each data point:

13 $$ c_{i} = \mathop {\arg \min }\limits_{j = 1,2, \ldots ,T} \, dist(x_{j} ,\mu_{i} ) $$

where $\arg \min$ represents the index of the minimum value.

*Step 4*: Introduce interpretable criterion 1 to ensure that the cluster centroids are updated within a reasonable range and that the updated centroids still maintain distinctiveness. The formula for updating the centroids is as follows:

14 $$ \begin{gathered} A_{i}^{k} = \mu_{i} = \frac{1}{{\left| {c_{i} } \right|}}\sum\limits_{{x_{i} \in c_{i} }} {x_{i} } \hfill \\ Criterion \, 1: \hfill \\ A_{i}^{k} \sim Q_{i}^{k} (k = 1,2, \cdots ,L) \hfill \\ s.t. \hfill \\ Q_{k} \in \{ \{ RA_{i}^{k} \_Min \le A_{i}^{k} \le RA_{i}^{k} \_Max\} \} \hfill \\ h \in \{ \{ A_{1}^{k} < A_{2}^{k} < \cdots < A_{i}^{k} \} \hfill \\ or\{ A_{1}^{k} > A_{2}^{k} > \cdots > A_{i}^{k} \} \} \hfill \\ \end{gathered} $$

*Step 5*: The objective function is the sum of squared errors within clusters, which is minimized:

15 $$ J = \sum\limits_{i = 1}^{T} {\sum\limits_{{x_{j} \in c_{i} }} {dist(x_{j} ,\mu_{i} )^{2} } } $$

where $J$ represents the sum of squares of errors in the cluster.

Repeat steps 2 to 5 until a certain criterion is met or the maximum number of iterations is reached. At this point, the obtained cluster centroids represent the optimized reference values, as shown in the following formula:

16 $$ \begin{gathered} h = \{ \mu_{1} ,\mu_{2} , \cdots ,\mu_{i} \} = \{ A_{1}^{k} ,A_{2}^{k} , \cdots ,A_{i}^{k} \} \hfill \\ i \in \{ 1,2, \cdots ,T\} \hfill \\ \end{gathered} $$

### Reference value optimized BRB

**Figure 4:**
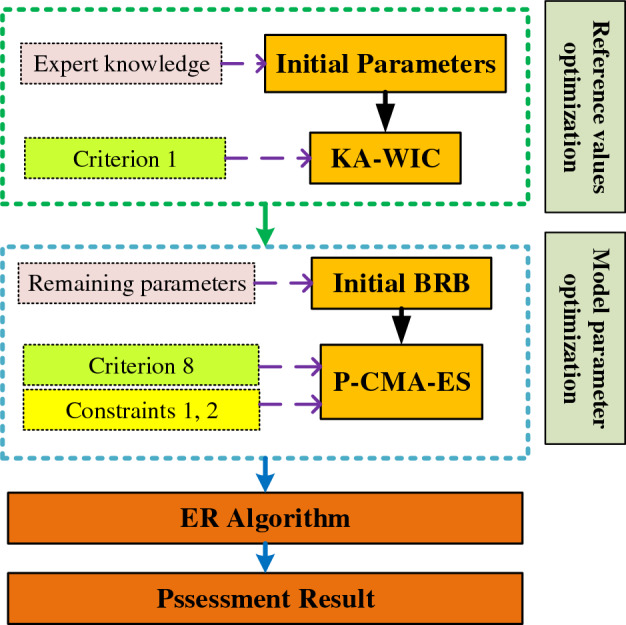
The modeling process of complex system health state assessment model based on I-BRB.

To address the challenges in complex system health assessment, an I-BRB model is constructed, where the $k_{th}$ rule is formulated as follows:

17 $$ \begin{gathered} R_{k} : \hfill \\ if \, x_{1} \, is \, A_{1}^{k} \wedge \, x_{2} \, is \, A_{2}^{k} \wedge \cdots \wedge \, x_{T} \, is \, A_{T}^{k} \hfill \\ then\{ (D_{1} ,\beta_{1,k} ),(D_{2} ,\beta_{2,k} ), \cdots ,(D_{N} ,\beta_{N,k} )\} ,\left( {\sum\limits_{n = 1}^{N} {\beta_{n,k} \le 1} } \right), \hfill \\ with \, rule \, weight \, \theta_{k} ,k\{ 1,2, \cdots ,L\} . \hfill \\ and \, attribute \, weight \, \delta_{1} ,\delta_{2} , \cdots \delta_{i} ,i \in \{ 1,2, \cdots ,W\} \hfill \\ in{\text{ C}}_{1} ,C_{2} , \cdots ,C_{n} \hfill \\ \end{gathered} $$

where ${\text{C}}_{1} ,C_{2} , \cdots ,C_{n}$ represents the interpretability constraints of the complex system health assessment model. The overall modeling process of I-BRB is illustrated in Fig. 4.

After constructing the I-BRB model for complex system health assessment, the inference process can be performed on each model. This process is based on the ER algorithm, and the inference process is transparent and interpretable^31^.

*Step 1*: Transforming different forms of information into belief distributions.

18 $$ S(d_{i} ) = \{ (A_{i,j} ,\alpha_{i,j} ),i = 1,...,M;j = 1,...,J_{i} \} $$

19 $$ a_{i,j}^{{}} = \left\{ \begin{gathered} \frac{{A_{i,j + 1} - x_{i} }}{{A_{i,j + 1} - A_{i,j} }}, \, j = k,if \, A_{i,j} \le x_{i} \le A_{i,j + 1} \hfill \\ \begin{array}{*{20}c} {\frac{{x_{i} - A_{i,j} }}{{A_{i,j + 1} - A_{i,j} }},{\text{ j}} = k + 1 \, } \\ {0,{\text{ j}} = 1,...,J_{i} ,j \ne k,k + 1} \\ \end{array} \hfill \\ \end{gathered} \right. $$

where $a_{i,j}$ represents the matching degree of the $i_{th}$ attribute and $A_{i,j}$ represents the corresponding reference values for that attribute.

*Step 2*: Calculate the activation weight $\omega_{k}$ for the $k_{th}$ rule using the following formula:

20 $$ \omega_{k} = \frac{{\theta_{k} \prod\limits_{i = 1}^{T} {\left( {a_{i,j}^{k} } \right)} \overline{{^{{\delta_{i} }} }} }}{{\sum\limits_{l = 1}^{M} {\theta_{l} } \prod\limits_{i = 1}^{T} {\left( {a_{i,j}^{l} } \right)} \overline{{^{{\delta_{i} }} }} }}{ , }\overline{{\delta_{i} }} = \frac{{\delta_{i} }}{{\mathop {max}\limits_{i = 1,...,T} \{ \delta_{i} \} }} $$

where $\overline{{\delta_{i} }}$ represents the attribute weight for the $i_{th}$ evaluation indicator.

*Step 3*: Generate the inference output belief degree $\beta_{n}$ using the ER algorithm.

21 $$ \beta_{n} = \frac{{\mu \times \left[ {\prod\limits_{k = 1}^{L} {\left( {\omega_{k} \beta_{n,k} + 1 - \omega_{k} \sum\limits_{j = 1}^{N} {\beta_{i,k} } } \right)} - \prod\limits_{k = 1}^{L} {\left( {1 - \omega_{k} \sum\limits_{j = 1}^{N} {\beta_{j,k} } } \right)} } \right]}}{{1 - \mu \times \left[ {\prod\limits_{k = 1}^{L} {\left( {1 - \omega_{k} } \right)} } \right]}} $$

22 $$ \mu = \left[ {\sum\limits_{n = 1}^{N} {\prod\limits_{k = 1}^{L} {\left( {\omega_{k} \beta_{n,k} + 1 - \omega_{k} \sum\limits_{j = 1}^{N} {\beta_{j,k} } } \right) - (N - 1)\prod\limits_{k = 1}^{L} {\left( {1 - \omega_{k} \sum\limits_{j = 1}^{N} {\beta_{j,k} } } \right)} } } } \right]^{ - 1} $$

*Step 4*: Calculate the expected utility value.

23 $$ S(A{\prime} ) = \{ (D_{n} ,\beta_{n} );n = 1,...,N\} $$

where $S\left( \cdot \right)$ represents the set of belief distributions, $A^{\prime}$ is the actual input vector.

### Optimization of remaining parameters

In the optimal case of reference values in BRB, the optimization of the remaining parameters, including rule weights, belief degrees, and attribute weights, is equally important. Even slight differences in these parameters can significantly affect the prediction accuracy of BRB^8^. In the current research stage, many high-performance algorithms are used for the optimization process of the model^29^. In this paper, the P-CMA-ES algorithm is employed to optimize the remaining parameters of I-BRB, further improving the model's accuracy. To ensure the interpretability of the model is not compromised during the optimization process, interpretability constraints 1, 2 and interpretability criterion 8 are embedded in the algorithm.

To optimize the remaining parameters of the model, including rule weights, belief degrees, and attribute weights, the objective function is formulated as follows:

24 $$ \begin{gathered} \min MSE(\Omega ) \hfill \\ s.t. \, \hfill \\ \Omega = \{ \theta_{k} ,\beta_{n,k} ,\delta_{k} \} \hfill \\ \sum\limits_{n = 1}^{N} {\beta_{{_{{_{n,k} }} }} } = 1 \, n \in \{ 1,...,N\} ,k \in \{ 1,2,...,W\} . \hfill \\ 0 \le \theta_{k} \le 1 \, k \in \{ 1,2,...,W\} . \hfill \\ 0 \le \delta_{i} \le 1 \, i \in \{ 1,2,...,T\} . \hfill \\ 0 \le \beta_{{_{{_{n,k} }} }} \le 1 \, n \in \{ 1,...,N\} ,k \in \{ 1,2,...,W\} . \hfill \\ \end{gathered} $$

where $MSE\left( \cdot \right)$ represents the prediction accuracy of the model, which can be further described as:

25 $$ MSE(\Omega ) = \frac{1}{M}\sum\limits_{m = 1}^{M} {\left( {output_{forecast} - out_{actual} } \right)^{2} } $$

where $M$ represents the number of samples, $output_{forecast}$ represents the model's predicted results, $out_{actual}$ represents the actual values.

**Figure 5:**
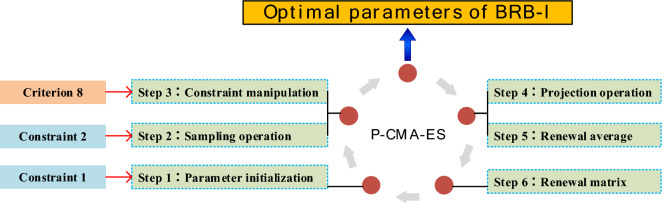
P-CMA-ES Algorithm with interpretability constraints.

The steps for running the P-CMA-ES algorithm are shown in Fig. 5, and the specific implementation process is as follows:

*Step 1*: To effectively utilize expert knowledge, incorporate interpretability constraint 1 during the parameter initialization step.

26 $$ {\text{Constraint 1}}: w^{g} = \left\{ {\begin{array}{*{20}c} {ek, \, if \, g = 1} \\ {w^{g} , \, if \, g \ne 1} \\ \end{array} } \right. $$

where the initial parameter set $w^{g} = \Omega^{0} (\theta ,\beta ,\delta )$ represents the parameters to be optimized. Interpretability constraint 1 incorporates expert knowledge into the initial population of the model, allowing expert knowledge to guide the optimization process and improve it. Additionally, interpretability constraint 1 ensures that the optimization starts near the optimal solution of the model.

*Step 2*: Sampling operation is performed to obtain each generation, incorporating interpretability constraint 2. The corresponding formula is as follows:

27 $$ \begin{gathered} \Omega_{i}^{(g + 1)} \sim H = w^{g} + \varepsilon^{g} N(0,C^{g} ) \hfill \\ {\text{Constraint 2:}} \hfill \\ H_{lp} \le H \le H_{up} :\{ \theta_{{lp_{k} }} \le \theta_{k} \le \theta_{{up_{k} }} \, k \in \{ 1,2, \ldots ,L\} . \hfill \\ \delta_{{lp_{i} }} \le \delta_{i} \le \delta_{{up_{i} }} \, n \in \{ 1, \ldots ,N\} . \hfill \\ \beta_{{lp_{k,n} }} \le \beta_{k,n} \le \beta_{{up_{k,n} }} \, i,n \in \{ 1,2, \ldots ,T\} \} . \hfill \\ \end{gathered} $$

28 $$ \Omega_{i}^{g + 1} \sim w^{g} + \varepsilon^{g} N(0,C^{g} ),i = 1, \ldots ,\lambda $$

where $\Omega_{i}^{g + 1}$ represents the $i_{th}$ solution in the $(g + 1)_{th}$ generation evolved, $w^{g}$ and $\varepsilon^{g}$ represent the strength generating and step size in generation $g_{th}$, $C^{g}$ denotes the covariance matrix of the strength generating in generation $g_{th}$, $N( * )$ and $\lambda$ represent the normal distribution and the number of offspring, respectively. Interpretability constraint 2 ensures that the parameters do not lose their physical meaning during the optimization process, thereby maintaining the interpretability of the model.

*Step 3*: Criterion operation, by using interpretability criterion 8, adjust the rules that are not consistent with reality.

29 $$ \begin{gathered} \Omega_{i}^{g + 1} \leftarrow \beta_{i}^{g + 1} = w^{g} + \varepsilon^{g} N(0,C^{g} ) \hfill \\ \beta_{i}^{g + 1} \sim C_{8} ,i = 1, \ldots ,\lambda \hfill \\ \end{gathered} $$

where $\Omega_{i}^{g + 1}$ represents the $i_{th}$ solution in the $g + 1_{th}$ generation, which may not be consistent with the actual belief distribution,$\beta_{i}^{g + 1}$ represents the reasonable belief generated under interpretability criterion 8, which is replaced through the $\leftarrow$ operation.

*Step 4*: Projection Operation: The solution is projected onto the feasible hyperplane to satisfy the constraint given by Eq. (30). The hyperplane can be represented by Eq. (31).

30 $$ \begin{gathered} \, \Omega_{i}^{g + 1} (1 + n_{e} \times (j - 1):n_{e} \times j) \hfill \\ = \, \Omega_{i}^{g + 1} (1 + n_{e} \times (j - 1):n_{e} \times j) \hfill \\ \quad - A_{e}^{T} \times (A_{e} \times A_{e}^{T} )^{ - 1} \times \Omega_{i}^{g + 1} (1 + n_{e} \times (j - 1):n_{e} \times j) \times A_{e} \hfill \\ \end{gathered} $$

31 $$ A_{e} \Omega_{i}^{g} (1 + n_{e} \times (j - 1):n_{e} \times j) = 1 $$

where $A_{e}$ represents the parameter vector, in the solution $\Omega_{i}^{g}$, $n_{e}$ and $j$ respectively denote the number of constrained variables and the number of equality constraints.

*Step 5*: Updating the mean of the next generation is done using the following formula:

32 $$ w^{g + 1} = \sum\limits_{i = 1}^{\tau } {h_{i} \Omega_{i:\lambda }^{g + 1} } $$

where $h_{i}$ represents the weight coefficient, $\Omega_{i:\lambda }^{g + 1}$ is the $i_{th}$ solution in the $\lambda$ solutions of the $(g + 1)_{th}$ generation, $\tau$ represents the size of the offspring population.

*Step 6*: The update formula for the covariance matrix is as follows:

33 $$ \begin{gathered} C^{g + 1} = (1 - c_{1} - c_{2} )C^{g} + c_{1} P_{c}^{g + 1} (P_{c}^{g + 1} )^{T} \hfill \\ \quad + c_{2} \sum\limits_{i = 1}^{v} {h_{i} } \left(\frac{{K_{i:\lambda }^{g + 1} - \varphi^{g} }}{{\rho^{g} }}\right)\left(\frac{{K_{i:\lambda }^{g + 1} - \varphi^{g} }}{{\rho^{g} }}\right)^{T} \hfill \\ \end{gathered} $$

where $\rho^{g}$ represents the step size of the $g_{th}$ generation, $c_{1}$ and $c_{2}$ represent the learning rates, $P_{c}^{g + 1}$ represents the evolution path of the $(g + 1)_{th}$ generation, $\varphi^{g}$ represents the offspring population size of the $g_{th}$ generation, $K_{i:\lambda }^{g + 1}$ represents the $i_{th}$ parameter vector of the $\lambda$ vector in the $(g + 1)_{th}$ generation.

*Step 7*: Recursively execute steps 1 to 6 until the best parameters are obtained.

## Case study

The flywheel system is a typical complex system, and its stable operation has a significant impact on the safe operation of spacecraft in orbit. Due to the high cost of conducting experiments on the entire flywheel system and the high failure rate of bearing components, this experiment only selects the flywheel bearing component as a case to validate the effectiveness of the proposed method. In this case, the elevated bearing temperature and decreased rotational speed are taken as two input indicators, and the bearing health status is the output.

The remaining parts of this section are arranged as follows: In Section "Initial I-BRB build", the optimization of reference values and the construction of the initial I-BRB model are discussed. In Section "Model optimization", the inference and optimization of the model are presented. In Section "Analysis of experimental results", the experimental results of the case study are analyzed. In Section "Contrast experiment", comparative experiments are discussed.

### Initial I-BRB build

In the BRB-based health assessment of complex systems, the reference values are initially provided by experts. Expert knowledge is accumulated knowledge of the long-term operation of the actual flywheel system and is an important source of interpretability for the BRB expert system. In this experiment, the dataset contains a total of 199 samples. 30% of the data is selected for model training, and 70% of the data is used for validation. The experts have set 4 reference values for each input indicator, as shown in Table 1, resulting in a total of 16 rules being defined^29^.

**Table 1:** Reference points and reference values.

	1	2	3	4
Z1	0	0.2	0.4	1.0
Z2	0	0.4	0.7	1.0
H	0.06	0.4	0.8	1.0

Among them, Z1 represents axial temperature, Z2 represents rotational speed, and H represents the health status of the bearing component. In this experiment, the health status is categorized into four levels: very poor (H1), poor (H2), fair (H3), and very good (H4). Due to the limitations of expert knowledge, the reference values provided by experts may not be sufficiently accurate. Therefore, it is necessary to optimize the reference values within a reasonable range in practical health assessment to improve the accuracy of model evaluation.

**Figure 6:**
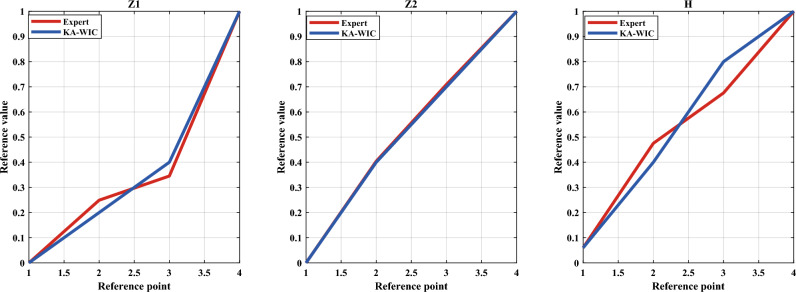
Reference values for I-BRB.

Under the constraint of interpretability criterion 1, the KA-WIC algorithm is employed to optimize the reference values. The reference points and reference value constraints are shown in Table 2, and the optimized results are presented in Fig. 6. In Fig. 6, the optimized reference values for Z1 (axial temperature) closely match the expert knowledge, while the optimized reference values for Z2 (rotational speed) are generally consistent with the expert knowledge. This indicates that the optimized reference values by the KA-WIC algorithm are locally optimized based on expert knowledge, fine-tuning them without compromising interpretability.

**Table 2:** Reference points and reference value constraints.

	1	2	3	4
Z1	0 ~ 0.1	0.1 ~ 0.30	0.30 ~ 1.0	1.0 ~ 1.2
Z2	0 ~ 0.1	0.1 ~ 0.7	0.7 ~ 1.0	1.0 ~ 1.2
H	0 ~ 0.06	0.06 ~ 0.6	0.6 ~ 1.0	0.95 ~ 1.2

To construct an interpretable model for the health assessment of the bearing components in the flywheel system, the remaining parameters of this experiment are also provided by experts. These parameters include attribute weights, rule weights, belief degrees, initial values, and interpretability constraints, as shown in Tables 3, 4 and 5.

**Table 3:** Initial attribute weights and constraints.

NO	The initial attribute weights	The attribute weights constraint
1	1	0.6 ~ 0.8
2	1	0.6 ~ 0.8

**Table 4:** Initial rule weights and constraints.

NO	The initial rule weights	The rule weights constraint
1	1	0.4 ~ 1
2	1	0.4 ~ 1
3	1	0.4 ~ 1
4	1	0.4 ~ 1
5	1	0.4 ~ 1
6	1	0.4 ~ 1
7	1	0.4 ~ 1
8	1	0.4 ~ 1
9	1	0.4 ~ 1
10	1	0.4 ~ 1
11	1	0.4 ~ 1
12	1	0.4 ~ 1
13	1	0.4 ~ 1
14	1	0.4 ~ 1
15	1	0 ~ 1
16	1	0 ~ 1

**Table 5:** Initial belief and constraints.

NO	The initial belief	The belief constraint
	$\left\{ {\beta_{1} ,\beta_{2} ,\beta_{3} ,\beta_{4} } \right\}$	$\left\{ {\beta_{1} ,\beta_{2} ,\beta_{3} ,\beta_{4} } \right\}$
1	{0.70, 0.30, 0.00, 0.00}	{0.65 ~ 0.70, 0.25 ~ 0.30, 0.00 ~ 0.05, 0.00 ~ 0.05}
2	{0.20, 0.70, 0.10, 0.00}	{0.15 ~ 0.20, 0.65 ~ 0.70, 0.05 ~ 0.10, 0.00 ~ 0.005}
3	{0.12, 0.13, 0.15, 0.60}	{0.10 ~ 0.15, 0.10 ~ 0.15, 0.15 ~ 0.20, 0.55 ~ 0.60}
4	{0.00, 0.00, 0.04, 0.96}	{0.00 ~ 0.05, 0.00 ~ 0.05, 0.00 ~ 0.05, 0.95 ~ 1.00}
5	{0.70, 0.10, 0.10, 0.10}	{0.70 ~ 0.75, 0.10 ~ 0.15, 0.10 ~ 0.15, 0.10 ~ 0.15}
6	{0.10, 0.40, 0.40,0.10}	{0.05 ~ 0.10, 0.40 ~ 0.45, 0.40 ~ 0.45, 0.05 ~ 0.10}
7	{0.03, 0.17, 0.40,0.40}	{0.00 ~ 0.05, 0.15 ~ 0.20, 0.35 ~ 0.40, 0.40 ~ 0.45}
8	{0.00, 0.00, 0.01, 0.99}	{0.00 ~ 0.05, 0.00 ~ 0.05, 0.00 ~ 0.05, 0.98 ~ 1.00}
9	{0.52, 0.19, 0.15, 0.14}	{0.50 ~ 0.55, 0.15 ~ 0.20, 0.10 ~ 0.15, 0.10 ~ 0.15}
10	{0.01, 0.57, 0.24, 0.18}	{0.00 ~ 0.05, 0.55 ~ 0.60, 0.20 ~ 0.25, 0.15 ~ 0.20}
11	{0.05, 0.07, 0.36, 0.52}	{0.00 ~ 0.05, 0.05 ~ 0.10, 0.35 ~ 0.40, 0.50 ~ 0.55}
12	{0.02, 0.02, 0.02, 0.94}	{0.00 ~ 0.05, 0.00 ~ 0.05, 0.00 ~ 0.05, 0.90 ~ 0.95}
13	{0.00, 0.00, 0.00, 1.00}	{0.00 ~ 0.05, 0.00 ~ 0.05, 0.00 ~ 0.05, 0.95 ~ 1.00}
14	{0.01, 0.02, 0.03, 0.94}	{0.00 ~ 0.05, 0.00 ~ 0.05, 0.00 ~ 0.05, 0.94 ~ 1.00}
15	{0.23, 0.33, 0.35, 0.09}	{0.05 ~ 0.10, 0.40 ~ 0.45, 0.40 ~ 0.45, 0.05 ~ 0.10}
16	{0.00, 0.00, 0.16, 0.84}	{0.00 ~ 0.01, 0.00 ~ 0.01, 0.15 ~ 0.20, 0.80 ~ 0.85}

To ensure that the optimization process improves accuracy without deviating from rationality, experts analyzed the overall belief distribution of the flywheel under different states based on the full-life operation analysis of multiple batches of the same model flywheels. This analysis was combined with in-orbit usage and historical failure cases. In the experimental case, there is a positive correlation between the health status levels of the assessment indicators, namely, the axle temperature and the rotational speed, and the health status level of the bearing. For example, when the temperature is in state H1 and the speed is in state H1, both indicators are in the worst state, indicating the poorest initial health status of the bearing. Based on their expertise, the experts set the initial belief distribution as {0.95, 0.05, 0.00, 0.00}, where the belief for the "very poor" health status assessment is 0.95, for the "poor" health status assessment is 0.05, and for the "fair" and "very good" health status assessments is 0. Due to the fuzziness and incompleteness of cognition, the initial parameter distribution provided by experts may not be perfectly accurate, but it can still provide a relatively reasonable initial parameter distribution.

Combining the optimized reference values with the initial values of attribute weights, rule weights, and belief degrees provided by experts, an initial I-BRB model for the health assessment of the flywheel is constructed.

### Model optimization

**Figure 7:**
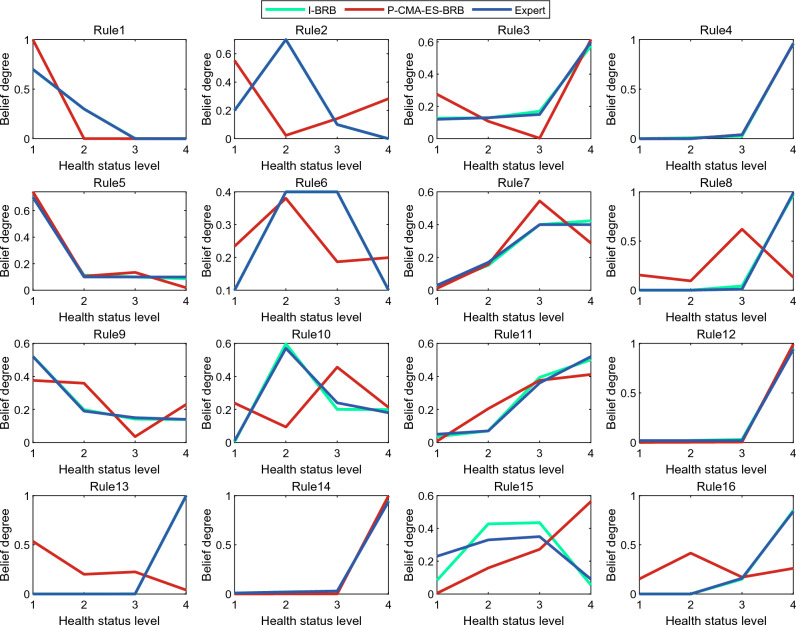
Belief comparison.

In the health assessment of complex systems, the initial parameters provided by experts may not be sufficiently accurate, which can affect the accuracy of the model. To improve the accuracy of the I-BRB model without compromising its interpretability, this experiment employs the P-CMA-ES algorithm with interpretability constraints 1, 2 and interpretability criterion 8 for model optimization. The optimized belief degrees are shown in Fig. 7.

Expert knowledge is an important source of interpretability for BRB-based complex systems, representing accumulated knowledge from the long-term operation of actual flywheel systems. Assuming that expert knowledge is authoritative and reliable, users can have a high level of trust in the initial BRB model constructed based on expert knowledge. By using expert knowledge as the initial input for belief distribution and appropriately adjusting it based on the data from the I-BRB model, the resulting belief distribution should not deviate excessively from the initially set distribution. The degree of proximity between the output belief distribution and the initial belief distribution can reflect the interpretability of the model. Therefore, the closer the belief after real-time data correction by the I-BRB model is to expert knowledge, the stronger the model's interpretability.

Due to the high reliability requirements for evaluation results in complex systems, experts are cautious when setting belief constraints and limit them to a relatively small range. In Fig. 7, it can be observed that for rules 1, 2, 3, 4, 5, 6, 9, 12, 13, 14, and 16, the optimized belief degrees of the I-BRB model are very close to the expert-provided initial reference values. This indicates that the P-CMA-ES algorithm with interpretability constraints can fine-tune the belief degrees to improve the accuracy of the model evaluation. Additionally, the evaluation results generated by these rules can be trusted by experts. For rules 7, 8, 10, 11, and 15, the belief degrees are close to the expert-provided belief distribution. This demonstrates that the I-BRB model can improve the accuracy of the model while maintaining interpretability. Therefore, the I-BRB model can be applied to the health assessment of complex systems.

In comparison, the flywheel health assessment model based on the KA-WIC algorithm and the BRB model optimized by the P-CMA-ES algorithm without interpretability constraints (K-P-BRB) yield less convincing evaluation results. The randomness of the P-CMA-ES algorithm in optimizing belief degrees can undermine the interpretability of the model. For example, in rules 2, 3, 5, 6, 8, 9, 10, 13, and 16, the distribution of belief degrees is concave or non-monotonic. Clearly, the evaluation results generated by these rules conflict with reality. In rules 1, 7, 11, and 15, there is a significant discrepancy between the optimized belief degrees and the expert-provided initial belief degrees. Only the evaluation results generated by rules 4, 12, and 14 can be accepted by decision-makers. Therefore, the K-P-BRB model is not suitable for the health assessment of complex systems. The optimized belief degrees, attribute weights, and rule weights are provided in the Tables 6 and 7.

**Table 6:** Belief and rule weights after I-BRB optimization.

No	Rule weight	The optimized belief
		$$\left\{ {\beta_{1} ,\beta_{2} ,\beta_{3} ,\beta_{4} } \right\}$$
1	0.885863000000000	{0.69, 0.29, 0.01, 0.01}
2	0.806674000000000	{0.20, 0.70, 0.09, 0.01}
3	0.754998000000000	{0.12, 0.13, 0.17, 0.58}
4	0.513410000000000	{0.01, 0.01, 0.02, 0.96}
5	0.747391000000000	{0.71, 0.12, 0.09, 0.08}
6	0.745770000000000	{0.09, 0.40, 0.40, 0.11}
7	0.782411000000000	{0.02, 0.15, 0.40, 0.43}
8	0.615898000000000	{0, 0, 0.04, 0.96}
9	0.931861000000000	{0.53, 0.19, 0.15, 0.13}
10	0.747368000000000	{0.01, 0.59, 0.21, 0.19}
11	0.636682000000000	{0.04, 0.07, 0.39, 0.50}
12	0.682577000000000	{0.01, 0.01, 0.03, 0.95}
13	0.457718000000000	{0, 0, 0.01, 0.99}
14	0.838830000000000	{0.01, 0.02, 0.02, 0.95}
15	7.200000000000e-05	{0.08, 0.42, 0.43, 0.05}
16	0.927711000000000	{0, 0.01, 0.15, 0.84}

**Table 7:** Optimized attribute weights.

NO	The attribute weights
1	0.629873895
2	0.731569947

### Analysis of experimental results

**Figure 8:**
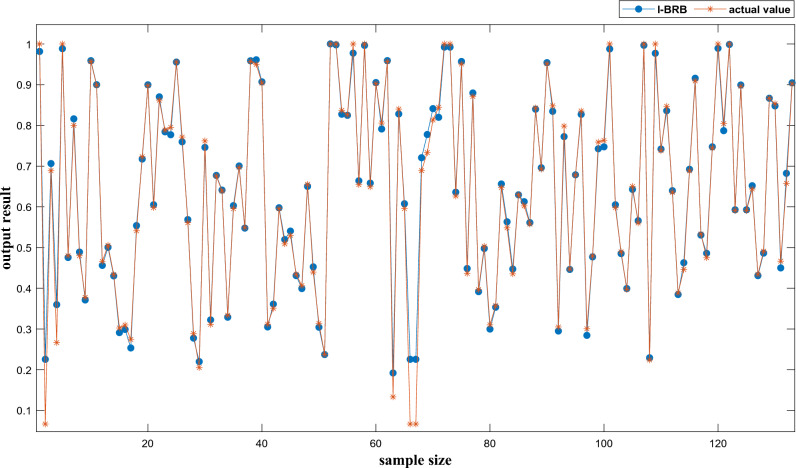
I-BRB evaluation results and actual values.

Based on the optimized I-BRB model for flywheel health state assessment, the ER algorithm was used to perform inference on the model. The comparison between the evaluation results of I-BRB and the actual values is shown in Fig. 8.

In Fig. 8, it can be observed that the evaluation results of I-BRB show a good fit with the actual values, indicating that I-BRB is capable of maintaining high accuracy while preserving interpretability.

### Contrast experiment

The complex system health assessment method, called P-BRB, is established by optimizing BRB using the P-CMA-ES algorithm without incorporating interpretability constraints. In this paper, various models including I-BRB, K-P-BRB, P-BRB, Linear Regression (LR), Robust Linear Regression (RLR), Decision Tree (DT), Medium Decision Tree (MDT), Coarse Decision Tree (CDT), Linear Support Vector Machine (LSVM), Fine Gaussian Process (FGP), Coarse Gaussian Process (CGP), Gradient Boosting Tree (GBT), and Random Forest (RF) are constructed for the assessment of the flywheel health status. The mean squared error (MSE) of the evaluation results is presented in Table 8.

**Table 8:** Comparative experiments of different models.

Methods	MSE
I-BRB	0.0007820
K-P-BRB	0.000952
P-BRB	0.007036
LR	0.0023653
RLR	0.0024522
DT	0.0091415
MDT	0.012876
CDT	0.019549
LSVM	0.0023577
FGP	0.0040586
CGP	0.00069358
GBT	0.0059844
RF	0.0053254

Compared to machine learning algorithms such as LR, RLR, DT, MDT, CDT, LSVM, FGP, GBT, and RF, I-BRB demonstrates better predictive accuracy and interpretability in the assessment of flywheel health status. Although CGP achieves higher predictive accuracy, its evaluation results lack interpretability and are difficult to convince decision-makers.

K-P-BRB shows significantly higher accuracy compared to P-BRB, indicating that the KA-WIC algorithm effectively adjusts the reference values and improves the model's accuracy. I-BRB, compared to K-P-BRB and P-BRB, achieves higher accuracy while maintaining interpretability.

Based on the above comparisons, I-BRB can be effectively applied to complex system health assessment problems. It improves modeling accuracy while retaining the interpretability of the model.

## Conclusion

In conclusion, this method provides a powerful approach for the health assessment of complex systems by conducting a comprehensive optimization of all parameters while preserving the interpretability of the BRB. By optimizing the reference values within a reasonable range, the method achieves improved accuracy while maintaining model interpretability.

The results demonstrate that the optimized reference values closely align with expert knowledge, indicating the effectiveness of the KA-WIC algorithm and P-CMA-ES algorithm in fine-tuning the reference values. The assessment model based on the optimized reference values outperforms machine learning algorithms such as LR, RLR, DT, MDT, CDT, LSVM, FGP, GBT, and RF in terms of both prediction accuracy and interpretability.

Furthermore, the I-BRB model surpasses the K-P-BRB and P-BRB models in accuracy and interpretability, highlighting its superiority in complex system health assessment. The CGP model exhibits higher prediction accuracy, but its lack of interpretability hinders its acceptance by decision-makers.

Overall, the proposed method, with its emphasis on reference value optimization and interpretability, offers an effective solution for complex system health assessment. It balances accuracy and comprehensibility, providing decision-makers with reliable and understandable assessment results. Future research can explore further enhancements to this method and its application in various domains to improve system reliability and decision-making processes.

## Data Availability

The datasets analysed in this study are not publicly available due to the unpublished intellectual property rights associated with the data, but are available on request from the corresponding authors.
